# Molecular Subtyping Based on Cuproptosis-Related Genes and Characterization of Tumor Microenvironment Infiltration in Kidney Renal Clear Cell Carcinoma

**DOI:** 10.3389/fonc.2022.919083

**Published:** 2022-07-06

**Authors:** Zhong-Hao Ji, Wen-Zhi Ren, Hao-Qi Wang, Wei Gao, Bao Yuan

**Affiliations:** ^1^ Department of Laboratory Animals, College of Animal Sciences, Jilin University, Changchun, China; ^2^ Department of Basic Medicine, Changzhi Medical College, Changzhi, China

**Keywords:** kidney renal clear cell carcinoma (KIRC), cuproptosis, gene signature, overall survival, immune status

## Abstract

The incidence of kidney renal clear cell carcinoma (KIRC) is rising worldwide, and the prognosis is poor. Cuproptosis is a new form of cell death that is dependent on and regulated by copper ions. The relationship between cuproptosis and KIRC remains unclear. In the current study, changes in cuproptosis-related genes (CRGs) in TCGA-KIRC transcriptional datasets were characterized, and the expression patterns of these genes were analyzed. We identified three main molecular subtypes and discovered that multilayer CRG changes were associated with patient clinicopathological traits, prognosis, elesclomol sensitivity, and tumor microenvironment (TME) cell infiltration characteristics. Then, a CRG score was created to predict overall survival (OS). The CRG score was found to be strongly linked to the TME. These findings may help elucidate the roles of CRGs in KIRC, potentially enhancing understanding of cuproptosis and supporting the development of more effective immunotherapy strategies.

## Introduction

In renal cell carcinoma, malignant tumors develop from renal tubular epithelial cells. This cancer accounts for almost 90% of renal malignancies and 3% of adult malignancies (1). Kidney renal clear cell carcinoma (KIRC) is the most common form of renal cell carcinoma (2). It is frequently asymptomatic, and approximately 30% of patients are diagnosed at an advanced stage. Because of its intrinsic resistance to radiation and chemotherapy, advanced KIRC has an exceedingly dismal prognosis (3). Therefore, there is a need to identify new and useful biomarkers for early diagnosis and treatment.

Copper is a cofactor for essential enzymes and therefore plays crucial roles in biological activities in organisms ranging from bacteria and fungi to plants, animals, and humans. Copper is a trace element, and either deficiency or excess can cause diseases (4). Tsvetkov et al. discovered a novel copper-dependent controlled cell death mode that differs from known cell death mechanisms and named it cuproptosis. Twelve cuproptosis-related genes (CRGs) were also identified, of which eight were positively regulated (FDX1, LIAS, LIPT1, DLD, DLAT, PDHA1, PDHB, and ATP7B) and four were negatively regulated (MTF1, GLS, CDKN2A, and SLC31A1) (5). Elesclomol, a copper ion carrier drug, has been shown to have therapeutic effects in a variety of diseases (6–8). The FDX1 gene encodes a small iron-sulfur protein involved in steroid, vitamin D and bile acid metabolism that is a key molecule in cuproptosis (9). It has been shown that genetic polymorphisms in FDX1 are associated with IgA nephropathy in the Han Chinese population (10, 11). In addition, recent studies have suggested that the FDX1 gene may play important roles in diseases such as lung adenocarcinoma (12) and polycystic ovary syndrome (PCOS) (13).

KIRC prognosis has not yet been associated with CRG variants. As a result, examining the molecular features of CRGs may aid in elucidating the source of heterogeneity in KIRC.

In this study, we separated 530 KIRC samples into three cuproptosis-associated subtypes based on 12 CRGs and examined the differences in survival and immune infiltration among the subtypes. We also developed a scoring system for predicting overall survival (OS) and characterizing the KIRC immunological landscape. The results reveal that the cuproptosis score is an effective prognostic indicator.

## Materials and Methods

### KIRC Datasets

The Gene Expression Omnibus (GEO) (14), the Genotype-Tissue Expression (GTEx) database (15) and The Cancer Genome Atlas (TCGA) (16) were used to obtain gene expression and pertinent prognostic and clinicopathological data for KIRC. A total of four datasets were obtained, including three from the GEO (GSE12606, GSE53000, and GSE53757) and the TCGA-KIRC dataset. Among them, the GSE12606 dataset contains 3 cancer samples and 3 paired normal samples, the GSE53000 dataset contains 53 cancer samples and 6 normal samples, the GSE53757 dataset contains 72 cancer samples and 72 paired normal samples. All data analysis was performed with R (version 4.0.3) and R Bioconductor.

### Consensus Clustering Analysis of CRGs

The TCGA dataset (https://portal.gdc.com) was utilized to perform molecular typing of KIRC for 12 CRGs. RNA sequencing expression profiles (level 3) and relevant clinical information were downloaded from the TCGA dataset. Consistency analysis was performed using the ConsensusClusterPlus R package (v1.54.0) with a maximum number of clusters of 6, and 80% of the total sample was drawn 100 times. Clustering heatmaps were created in R using the package pheatmap (v1.0.12). Genes with a standard deviation over 0.1 were included in the gene expression heatmap.

### Relationship Among Molecular Subtypes and Clinical Characteristics, Prognosis, Elesclomol Sensitivity, Tumor Stemness, Immune Infiltration, and Immune Checkpoints in KIRC

We investigated the correlations among molecular subtypes, clinicopathological features, and prognosis to assess the clinical utility of the three subtypes. Kaplan–Meier (KM) curves produced by the survival and survminer R programs were used to analyze variations in OS among the various subtypes. Based on the Genomics of Drug Sensitivity in Cancer (GDSC) database, the pRRophetic R package was used to build the prediction procedure. The half-maximal inhibitory concentrations (IC50) of the samples were calculated using ridge regression (17).

The one-class logistic regression (OCLR) method was used to calculate an mRNA expression-based stemness index (mRNAsi) to assess tumor stemness (18). The relative percentages of 22 types of immune cells were evaluated using CIBERSORT (19) to ascertain the immunological features of the KIRC samples. Eight genes (SIGLEC15, TIGIT, CD274, HAVCR2, PDCD1, CTLA4, LAG3 and PDCD1LG2) were used for immune checkpoint analysis (20).

### Differentially Expressed Gene (DEG) Identification and Functional Annotation

Considering the limited sample size of the G3 subtype, subsequent analyses were performed between the G1 and G2 subtypes. Using the limma package in R, we identified DEGs between the various subtypes of cuproptosis according to thresholds of a fold-change (FC) of 2 and an adjusted P value of 0.05. We performed functional enrichment analyses of the DEGs using the clusterprofiler package in R to investigate the likely functions of the DEGs associated with each cuproptosis pattern.

### Construction of a Prognostic CRG Signature

By constructing a Venn diagram, we identified the common genes among the four datasets (G1/G2-DEGs, GSE12606, GSE53000, and GSE53757) and further analyzed the correlations between the expression of 14 DEGs and 12 CRGs. Using the glmnet R package, the LASSO Cox regression technique was utilized to minimize the risk of overfitting based on the 14 prognostic CRGs. To build the model, we performed tenfold cross-validation after examining each independent variable’s annual change. Finally, the cuproptosis gene signature, denoted as the CRG score ((Expi * coefi)), was constructed using 6 hub genes and their correlation coefficients. The surv cutpoint function from the survminer R package was used to calculate the appropriate cutoff value. On the basis of the best cutoff value of the score, 530 KIRC samples were grouped into high- and low-CRG score groups. The survminer R program was then used to perform survival analysis for the two groups. Finally, the area under the curve (AUC) values were determined by performing receive operating characteristic (ROC) analysis, and the signature’s predictive capabilities were assessed. Tumor-infiltrating immune cell analysis was performed using quanTIseq (21), EPIC (22), MCP-counter (23), and TIMER (24).

### Statistical Analyses

To perform statistical analysis, the R language (version 4.0.3) was used. KM survival analysis was performed along with a log-rank test to compare survival. Correlated variables without normal distributions were examined *via* Spearman’s correlation analysis. P<0.05 was considered to indicate a statistically significant difference.

## Results

### Transcriptional Alterations and Diagnostic Prognostic Value of CRGs in KIRC

**Figure 1:**
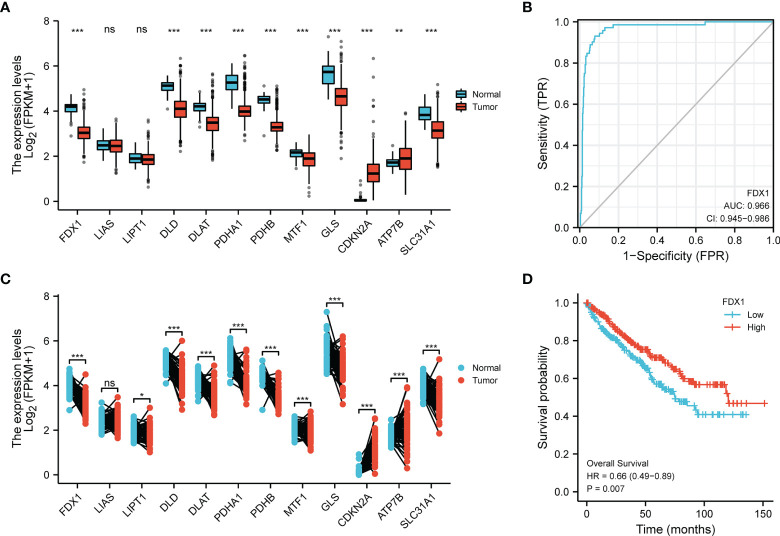
Transcriptional alterations and diagnostic/prognostic value of CRGs in KIRC. **(A)** Expression of 12 CRGs in tumors and normal tissues. **(B)** Expression of 12 CRGs in tumors and juxta-tumoral tissues. **(C)** ROC analysis of FDX1 as a diagnostic marker. **(D)** KM curves of OS. *p < 0.05, **p < 0.01, ***p < 0.001, ns, p > 0.05.

The expression of 12 CRGs in KIRC was analyzed using data from the TCGA and GTEx databases. The results showed that 10 genes were differentially expressed between tumor tissues and normal tissues (P < 0.01) (**Figure 1A**), while 11 genes were differentially expressed between paired tumors and juxta-tumoral tissues (P < 0.05) (**Figure 1B**). The diagnostic/prognostic value of FDX1 in KIRC was further analyzed, as FDX1 is a core molecule in cuproptosis. ROC analysis showed an AUC of 0.966, suggesting a good diagnostic efficacy of FDX1 in KIRC (**Figure 1C**). Patients with higher FDX1 expression had better survival rates (P=0.007) (**Figure 1D**). The complete ROC analysis results, KM analysis results and univariate Cox regression analysis results for the 12 CRGs are presented in **Figure S1**.

### Identification of Cuproptosis-Related Subtypes of KIRC

**Figure 2:**
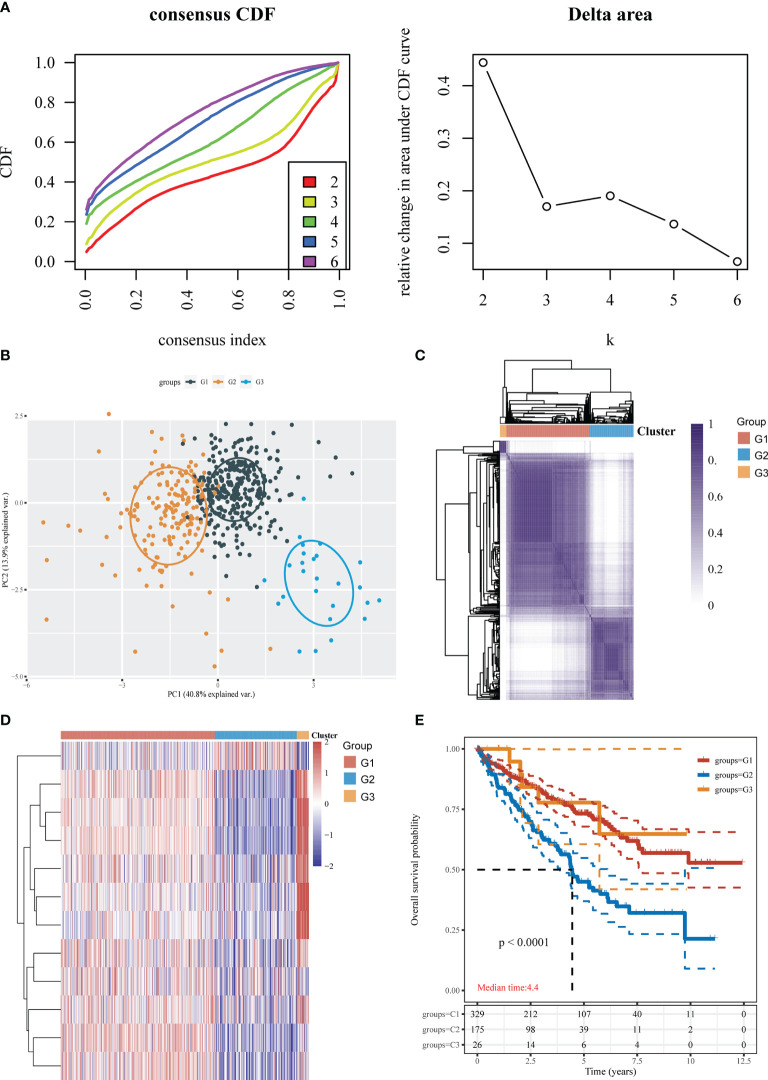
Identification of cuproptosis subtypes in KIRC. **(A)** Consensus clustering cumulative distribution function (CDF) and relative change in the area under the CDF curve (CDF delta area). **(B)** The obtained subtypes were subjected to PCA. **(C)** Three cluster subgroups. **(D)** Heatmap showing the DEGs among the 3 subtypes. **(E)** KM survival analysis of the different groups of samples from the TCGA dataset.

To better understand CRG expression in KIRC, we used consensus clustering to identify patients with KIRC based on the expression profiles of the 12 CRGs. Our results revealed that k = 3 was the best choice for categorizing the complete population; thus, the population was classified into the G1 (n = 329), G2 (n = 175), and G3 (n = 26) subtypes (**Figures 2A, C**; **Table S1**). Significant differences in the cuproptosis transcription profiles across the three subtypes were discovered using principal component analysis (PCA) (**Figure 2B**). The gene expression profiles of the three groups are shown in a heatmap (**Figure 2D**). As determined by the KM curves, patients with subtypes G1 and G3 had a longer OS than patients with subtype G2 (log-rank test, P<0.0001; **Figure 2E**).

### Characteristics of Elesclomol Sensitivity, Tumor Stemness, Immune Infiltration, Clinicopathological Features and Immune Checkpoints in the Cuproptosis-Related Subtypes

**Figure 3:**
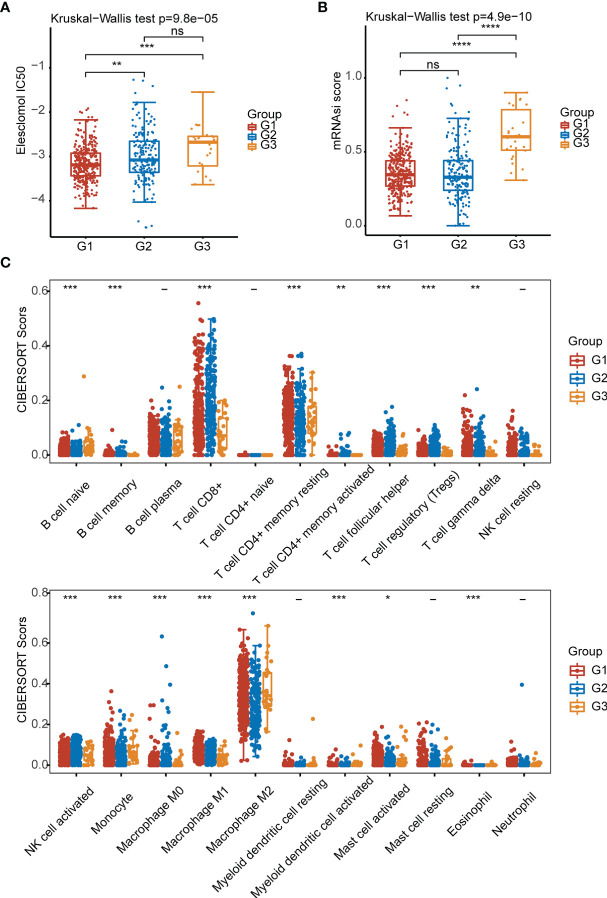
Elesclomol sensitivity, tumor stemness and immune infiltration characteristics in the three subtypes of KIRC. **(A)** IC50 values of elesclomol. **(B)** Tumor stemness scores. **(C)** Abundances of 22 infiltrating immune cell types in the three subtypes. *p < 0.05, **p < 0.01, ***p < 0.001, ****p < 0.0001, ns, p > 0.05.

Elesclomol is a highly lipophilic Cu^2+^-binding molecule that has been shown to induce cuproptosis (5). We tested the sensitivity of patients in the G1, G2, and G3 groups to elesclomol, which is already used as a chemotherapeutic medication for KIRC. The elesclomol IC50 of the G1 subtype was significantly lower than that of the G2 subtype (P<0.01) (**Figure 3A**). The cancer stem cell (CSC) index was lower in the G1 and G2 subtypes than in the G3 subtype according to the tumor stemness scores for the three subtypes (P < 0.001) (**Figure 3B**). To evaluate immune infiltration, we used the CIBERSORT algorithm. As shown in the bar graph, the cuproptosis-related subtypes were positively correlated with infiltration of naive B cells, memory B cells, CD8+ T cells, resting memory CD4+ T cells, activated memory CD4+ T cells, follicular helper T cells, regulatory T cells (Tregs), gamma delta T cells, activated natural killer (NK) cells, monocytes, M0 macrophages, M1 macrophages, M2 macrophages, activated myeloid dendritic cells, activated mast cells and eosinophils (**Figure 3C**).

**Figure 4:**
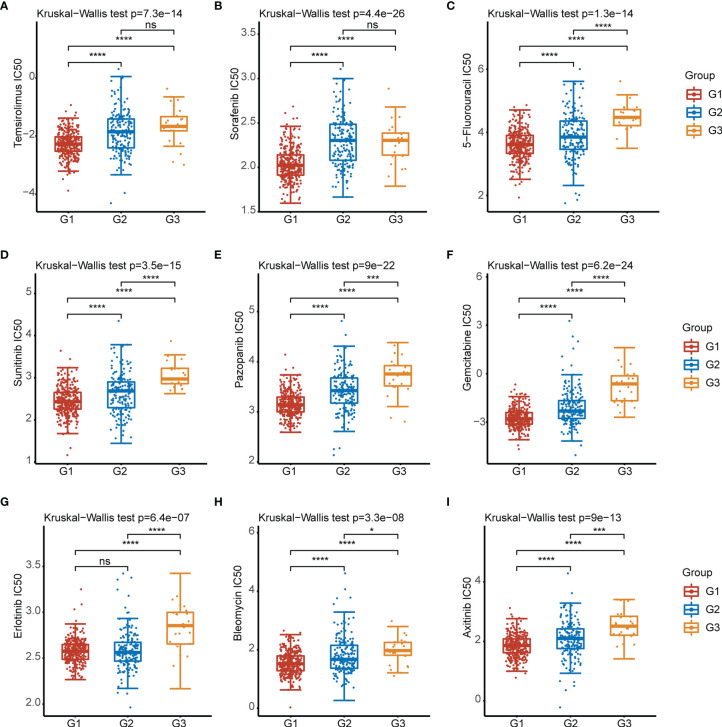
IC50 of 9 commonly used chemotherapeutic drugs and targeted drugs in the three subtypes of KIRC. **(A–I)** Temsirolimus, Sorafenib, 5-Fluorouracil, Sunitinib, Pazopanib,Gemcitabine, Erlotinib, Bleomycin, Axitinib. *p < 0.05, ***p < 0.001, ****p < 0.0001, ns, p > 0.05.

To elucidate the roles of CRGs on sensitivity to chemotherapeutics and targeted agents. We analyzed the sensitivity of G1, G2, and G3 groups of patients to nine kinds of commonly used chemotherapy and targeted drugs (Temsirolimus, Sorafenib, 5-Fluorouracil, Sunitinib, Pazopanib, Gemcitabine, Erlotinib, Bleomycin, Axitinib) for clear cell renal cell carcinoma. The results showed that the IC50 of 8 drugs (except erlotinib) in the G1 group were significantly lower than those in the G2 group (**Figure 4**).

**Figure 5:**
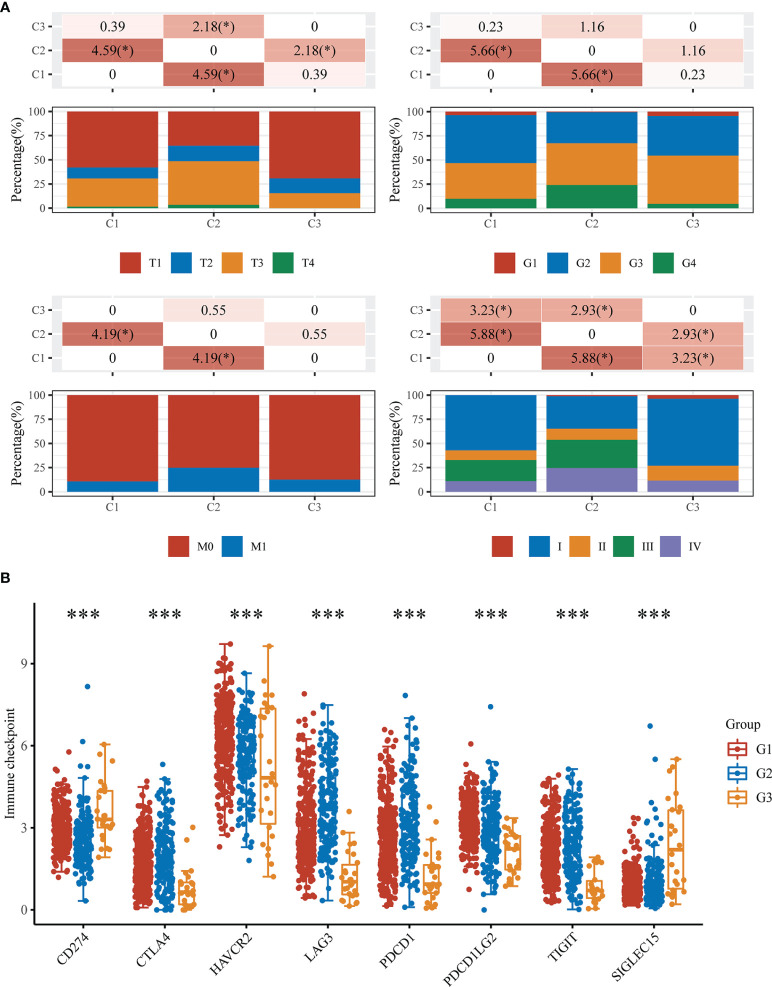
Clinicopathological and immune checkpoint characteristics in the three subtypes of KIRC. **(A)** Differences in clinicopathologic features among the three subtypes. **(B)** Expression levels of 8 immune checkpoint genes in the three subtypes. *p < 0.05, ***p < 0.001.

There was a significant difference in tumor stage between the G1 and G2 groups, while the T stage and total tumor stage were significantly different between the G2 and G3 groups (**Figure 5A**). Furthermore, when we investigated the correlations between immune checkpoints and subtypes of cuproptosis, we found that seven immune checkpoint proteins were differentially expressed among the three groups (**Figure 5B**).

### Comprehensive Analysis of Cuproptosis-Related DEGs in KIRC

**Figure 6:**
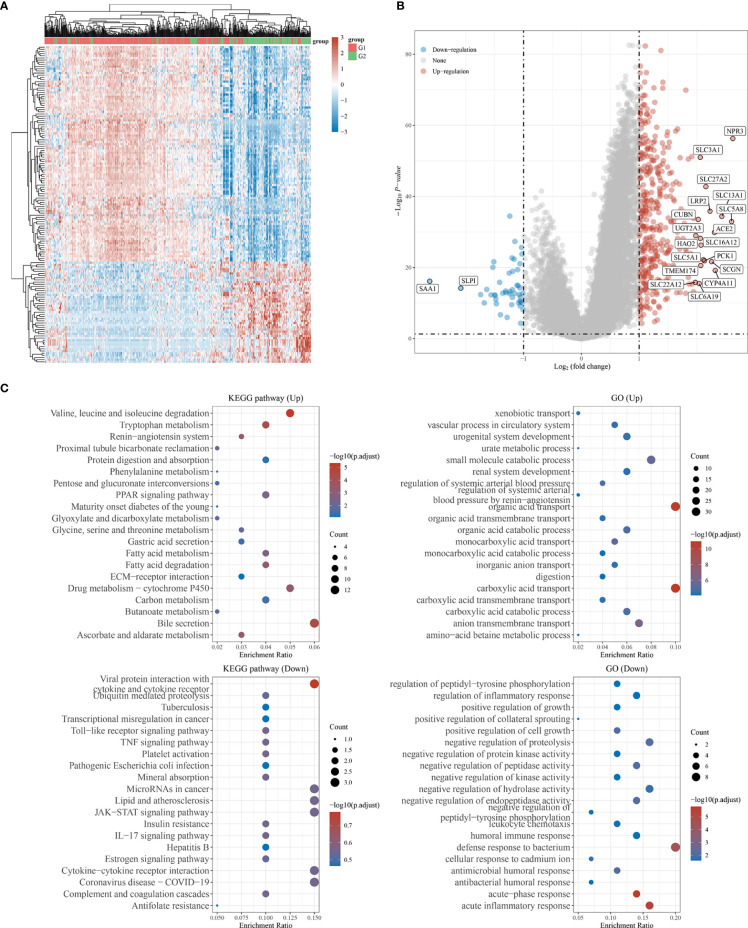
GO and KEGG analysis of the DEGs in between G1 subtype and G2 subtype. **(A)** Heat map showing the differential genes. **(B)** Volcano plot of differential gene expression analysis. **(C)** GO and KEGG enrichment analysis. (|LogFC|>1, Adjusted P <0.05).

Considering the limited sample size of the G3 subtype, subsequent analyses were performed between the G1 and G2 subtypes. To explore the potential biological functions of the cuproptosis-related subtypes of KIRC, we investigated the DEGs between the G1 and G2 subtypes with the limma R program, which generated 438 genes (**Table S2**). The heatmap and volcano map show the DEGs obtained from the G1/G2 comparison (**Figures 6A, B**); 391 genes were upregulated, while 47 genes were downregulated (|logFC|>1, adjusted P <0.05). According to GO and KEGG analyses, the functions of the DEGs were predominantly related to valine, leucine and isoleucine degradation; bile secretion; drug metabolism *via* cytochrome P450; and carboxylic acid transport (**Figure 6C**). The results of KM survival analysis for the 438 DEGs are shown in **Table S3**.

### Construction of the Prognostic CRG Score

**Figure 7:**
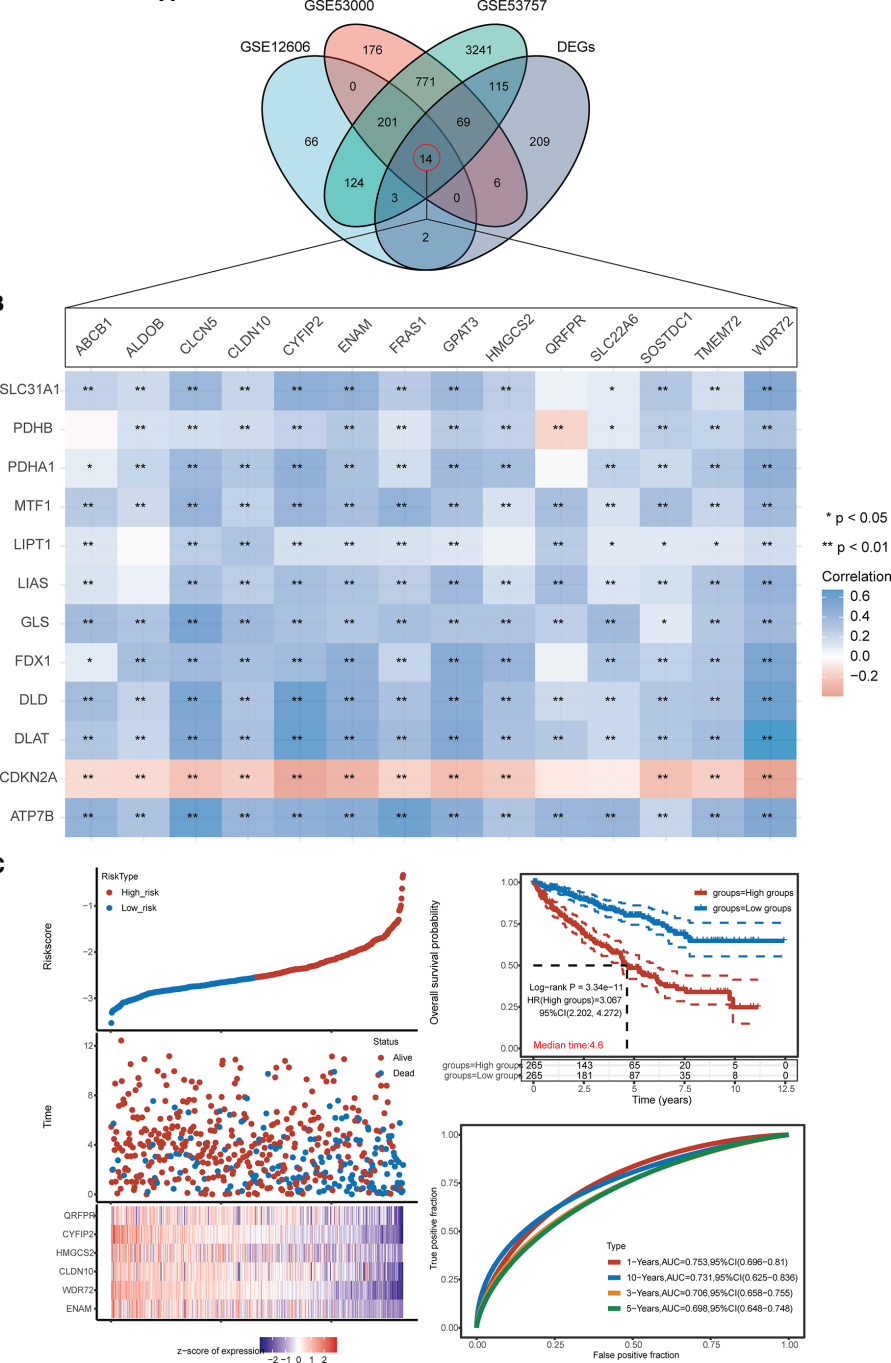
Construction of the prognostic CRG score. **(A)** VN map shows the overlap of 4 datasets (G1/G2-DEGs, GSE12606, GSE53000, GSE53757). **(B)** Correlation heat map of 14 intersection genes and 12 CRGs. **(C)** Ranked dot and scatter plots showing the CRG score distribution and patient survival status, Kaplan–Meier analysis of the OS between the two groups. (h) ROC curves to predict the sensitivity and specificity of 1-, 3-, 5-, and 10-year survival according to the CRG score. *p < 0.05, **p < 0.01.

Fourteen genes were shared among all four datasets (G1/G2-DEGs, GSE12606, GSE53000, and GSE53757) (**Figure 7A**; **Table S4**). Univariate Cox regression analysis revealed the 14 intersecting genes to be prognosis related, including QRFPR, CYFIP2, HMGCS2, CLDN10, WDR72, ENAM, ABCB1, ALDOB, CLCN5, FRAS1, GPAT3, SLC22A6, SOSTDC1, and TMEM72 (**Figure S2**). Heatmaps were then used to demonstrate the correlations between the expression of the 14 intersecting genes and the 12 CRGS (**Figure 7B**). Our prognostic model was developed based on the expression profiles of the 14 genes described above using LASSO Cox regression analysis, and a signature of 6 genes (ENAM, WDR72, CLDN10, HMGCS2, CYFIP2, and QRFPR) was discovered. According to survival analyses based on each gene’s ideal cutoff expression value, the high-risk group had a poor prognosis (P<0.05). Additionally, the 1-year, 3-year, 5-year, and 10-year survival rates predicted by the CRG score exhibited AUC values of 0.753, 0.706, 0.698, and 0.731, respectively (**Figure 7C**; **Figure S3**).

The CRG score was constructed as follows:


CRG score=(−0.006)*ENAM+(−0.1496)*WDR72+(−0.0583)*CLDN10+(−0.0019)*HMGCS2+(−0.217)*CYFIP2+(−0.0441)*QRFPR.

### The CRG Score Can Represent TME Differences

**Figure 8:**
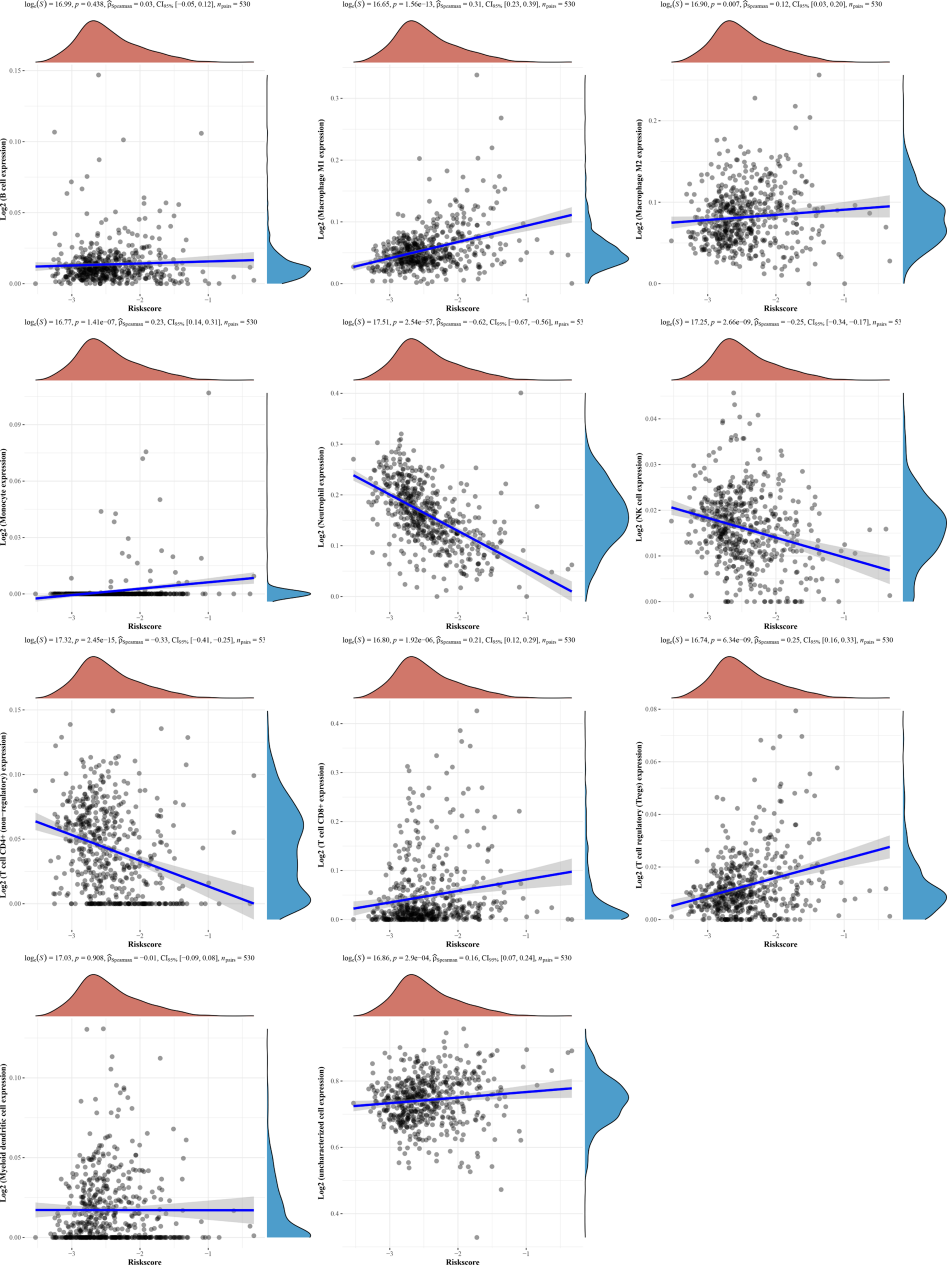
Evaluation of the TME between the two groups using the quanTIseq.

The results of the above analysis show that the diagnostic model we constructed, CRG score, can play an important role in clinical prediction. To evaluate whether the CRG score has guiding value for immunotherapy, the correlation of the CRG score with infiltration of various immune cells was further analyzed using four immune algorithms (quanTIseq, EPIC, MCP-counter, and TIMER). QuantTIseq-based analysis showed a positive correlation between the CRG score and infiltration of macrophages, monocytes, CD8+ T cells, and Tregs; however, a negative correlation was found between the CRG score and infiltration of neutrophils, NK cells, and nonregulatory CD4+ T cells. The Spearman correlation coefficient between the CRG score and neutrophil infiltration was -0.62 CRG score (**Figure 8**). The analysis results from the remaining three algorithms are shown in **Figure S4**.

## Discussion

KIRC is a common urological malignancy involving tumors. The insensitivity of KIRC to conventional radiotherapy and chemotherapy has made surgical resection the main treatment; however, distal metastases occur in nearly 40% of patients after surgery (25, 26). In addition, molecular heterogeneity affects the prognosis of cancer patients, so it is crucial to identify appropriate prognostic molecules (27).

Copper is a double-edged sword: it is a key cofactor for many enzymes, but excess copper ions can lead to cell death. The results of Peter Tsvetkov et al. suggest that copper ions regulate and control cuproptosis, a new form of cell death. During cuproptosis, copper ions directly bind to lipid-acylated components of the tricarboxylic acid cycle pathway, leading to abnormal aggregation of lipid-acylated proteins and loss of iron-sulfur cluster proteins and resulting in a proteotoxic stress response that leads to cell death (5). Kidney disease is closely linked to mitochondrial dysfunction (28–30), and inhibition of mitochondrial respiration significantly enhances the efficacy of chemotherapy for renal cell carcinoma (31). The use of copper ion carriers to kill cancer cells is a potential new treatment for cancer.

We examined the expression of 12 CRGs and the potential value of these genes as diagnostic/prognostic markers for KIRC. We found that 11 genes were differentially expressed in tumors and juxta-tumoral tissues, 6 genes had good diagnostic efficacy (AUC > 0.9), and 8 genes were associated with OS prognosis. Based on these results, we hypothesized that cuproptosis may play a role in the development of KIRC. To test this hypothesis, we examined 3 subtypes of KIRC using the 12 CRGs. Analysis of the sensitivity of the different subtypes to elesclomol, an anticancer drug known to mediate cuproptosis, showed that the G1 subtype with better OS prognosis was more sensitive than the G2 subtype. This finding confirmed the speculation that cuproptosis plays a role in KIRC. Further analysis of immune cell infiltration, clinical features, and immune checkpoints suggested that cuproptosis may influence the treatment and prognosis of KIRC by modulating the immune microenvironment. A comparison of 3 GEO datasets and the DEGs between G1 and G2 identified 14 genes. LASSO Cox regression was used to construct a prediction model with 6 genes (ENAM, WDR72, CLDN10, HMGCS2, CYFIP2, and QRFPR). In recent years, some researchers have analyzed the relationship between cell death-related phenotypes and KIRC, and established a corresponding prognostic diagnostic model. The 1-year AUC value of the ferroptosis-related gene diagnostic model was 0.733 (32). The 1-year AUC value of the Pyroptosis-related gene diagnosis model was 0.57 (33), and the 1-year AUC value of the Autophagy-related gene diagnosis model was 0.728 (34). In this study the 1-year survival rates predicted by the CRG score exhibited AUC values of 0.753. Therefore, the performance of our established diagnostic model is equivalent to or even better than some previous research reports.

ENAM and WDR72 gene polymorphisms are associated with enamel development (35, 36), and their mutations may lead to enamel hypoplasia (37–39). However, recent studies have found that the ENAM gene is associated with T classification and inhibits the proliferation of renal clear cell carcinoma (40) and that WDR72 genomic variants are associated with distal tubular acidosis and rapid declines in renal function (41, 42). CLDN10 is a member of the tight junction protein claudin family, and abnormal CLDN10 expression may lead to tight junction dysfunction and thus affect tumor progression. In Yang et al.’s study (43), CLDN10 associated with immune infiltration was found to be a novel prognostic biomarker for clear cell renal cell carcinoma. HMGCS2 functions as a ketogenic rate-limiting enzyme and plays oncogenic roles in hepatocellular carcinoma (44) and prostate cancer (45). CYFIP was initially widely considered a brain disease-associated protein (46), but recent studies have shown that its mutation and abnormal expression are associated with multiple cancers (47–49). In contrast, there have been relatively few studies on QRFPR, and the relationship of this gene with cancer is not very clear. Among the 6 genes for which we constructed a prognostic model, ENAM has been reported to be associated with immune infiltration and to exert oncogenic functions in KIRC, which reflects the reliability of our findings. The remaining 5 genes (WDR72, CLDN10, HMGCS2, CYFIP2, and QRFPR) have not been reported to be associated with KIRC but still have potential for exploration.

Notably, this study had many limitations. The stability of the subtyping strategy needs to be verified in a larger number of KIRC samples, and the relationship between cuproptosis and KIRC needs to be demonstrated experimentally. In addition, the underlying mechanisms between the CRGs and tumor immunity in KIRC are currently unknown and warrant further investigation.

## Conclusion

In conclusion, our study suggests that cuproptosis may be involved in the development of KIRC and defines a novel prognostic model with six genes. This model correlates with OS prognosis in TCGA data, providing insights into the prediction of KIRC prognosis and characterizing the KIRC immunological landscape.

## Data Availability Statement

The datasets analyzed for this study can be found in the TCGA-KIRC/READ project (http://www.cancer.gov/tcga) and GEO(https://www.ncbi.nlm.nih.gov/geo/query/acc.cgi?acc=GSE12606/GSE53000/GSE53757).

## Author Contributions

WG and BY were responsible for the main experimental concept and design; the analysis were performed by Z-HJ, W-ZR, and H-QW; and the manuscript was written by Z-HJ, W-ZR, WG, and BY. All of the authors approved the final version. All authors have read and agreed to the published version of the manuscript.

## Funding

This study was supported by the National Natural Science Foundation of China (32172729).

## Conflict of Interest

The authors declare that the research was conducted in the absence of any commercial or financial relationships that could be construed as a potential conflict of interest.

## Publisher’s Note

All claims expressed in this article are solely those of the authors and do not necessarily represent those of their affiliated organizations, or those of the publisher, the editors and the reviewers. Any product that may be evaluated in this article, or claim that may be made by its manufacturer, is not guaranteed or endorsed by the publisher.

## Abbreviations

KIRC, kidney renal clear cell carcinoma; LASSO, Least absolute shrinkage and selection operator; TCGA, The Cancer Genome Atlas; DEGs, Differentially expressed genes; OS, Overall survival; ROC, Receiver operating characteristic; GO, Gene Ontology; KEGG, Kyoto Encyclopedia of Genes and Genomes; PCA, Principal component analysis; AUC, Area under the curve; HR, Hazard ratio; GDSC, Genomics of Drug Sensitivity in Cancer; PCOS, Polycystic ovary syndrome; TME, Tumor Microenvironment; IC50, half-maximal inhibitory concentration.
