# Supplementary material for: Molecular Subtyping Based on Cuproptosis-Related Genes and Characterization of Tumor Microenvironment Infiltration in Kidney Renal Clear Cell Carcinoma
Source: Front Oncol. 2022 Jul 6;12:919083. doi: 10.3389/fonc.2022.919083 (PMC9299088; doi:10.3389/fonc.2022.919083)
Supplement: Supplementary file 1 [file Image_1.pdf]

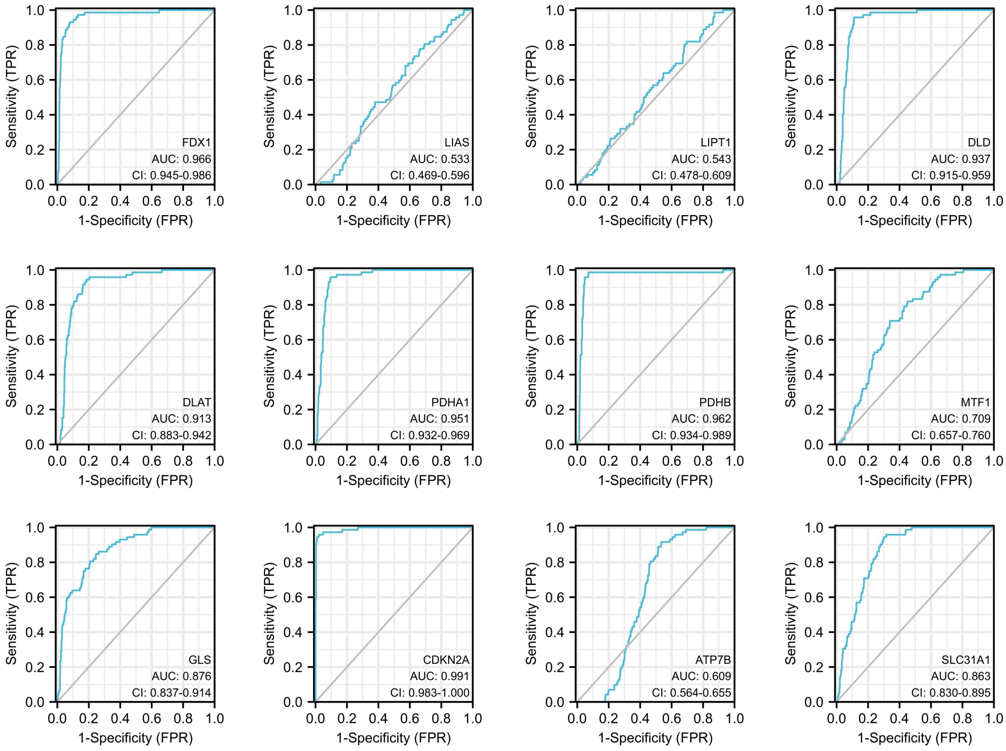

Fig.S1.1 Results of ROC analysis of 12 CRGS in KIRC.

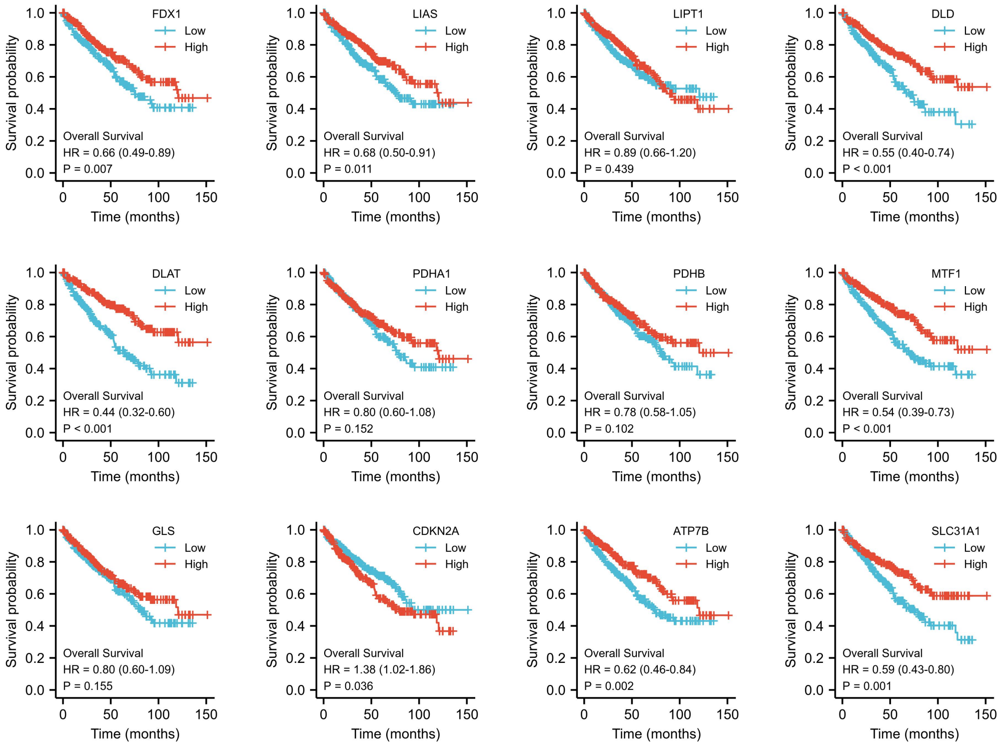

Fig.S1.2 Results of KM analysis of 12 CRGS in KIRC.

Characteristics	Total(N)	HR(95% CI) Univariate analysis	P value Univariate analysis
FDX1	539		
Low	269	Reference	
High	270	0.659 (0.487-0.891)	0.007
LIAS	539		
Low	269	Reference	
High	270	0.676 (0.500-0.915)	0.011
LIPT1	539		
Low	269	Reference	
High	270	0.889 (0.660-1.198)	0.439
DLD	539		
Low	269	Reference	
High	270	0.545 (0.402-0.741)	<0.001
DLAT	539		
Low	269	Reference	
High	270	0.435 (0.318-0.596)	<0.001
PDHA1	539		
Low	269	Reference	
High	270	0.803 (0.595-1.084)	0.152
PDHB	539		
Low	269	Reference	
High	270	0.778 (0.577-1.051)	0.102
MTF1	539		
Low	269	Reference	
High	270	0.535 (0.392-0.730)	<0.001
GLS	539		
Low	269	Reference	
High	270	0.805 (0.597-1.086)	0.155
CDKN2A	539		
Low	269	Reference	
High	270	1.379 (1.021-1.863)	0.036
ATP7B	539		
Low	269	Reference	
High	270	0.619 (0.456-0.840)	0.002
SLC31A1	539		
Low	269	Reference	
High	270	0.588 (0.432-0.800)	<0.001

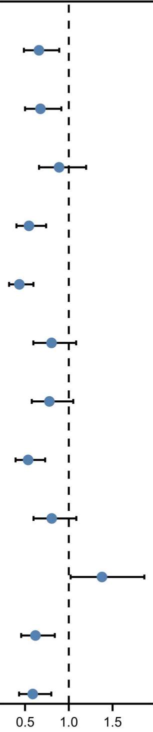

Fig.S1.3 Results of univariate Cox regression analysis of 12 CRGS in KIRC.
