# Supplementary figures and images for: Molecular Subtyping Based on Cuproptosis-Related Genes and Characterization of Tumor Microenvironment Infiltration in Kidney Renal Clear Cell Carcinoma
Source: Front Oncol. 2022 Jul 6;12:919083. doi: 10.3389/fonc.2022.919083 (PMC9299088; doi:10.3389/fonc.2022.919083)

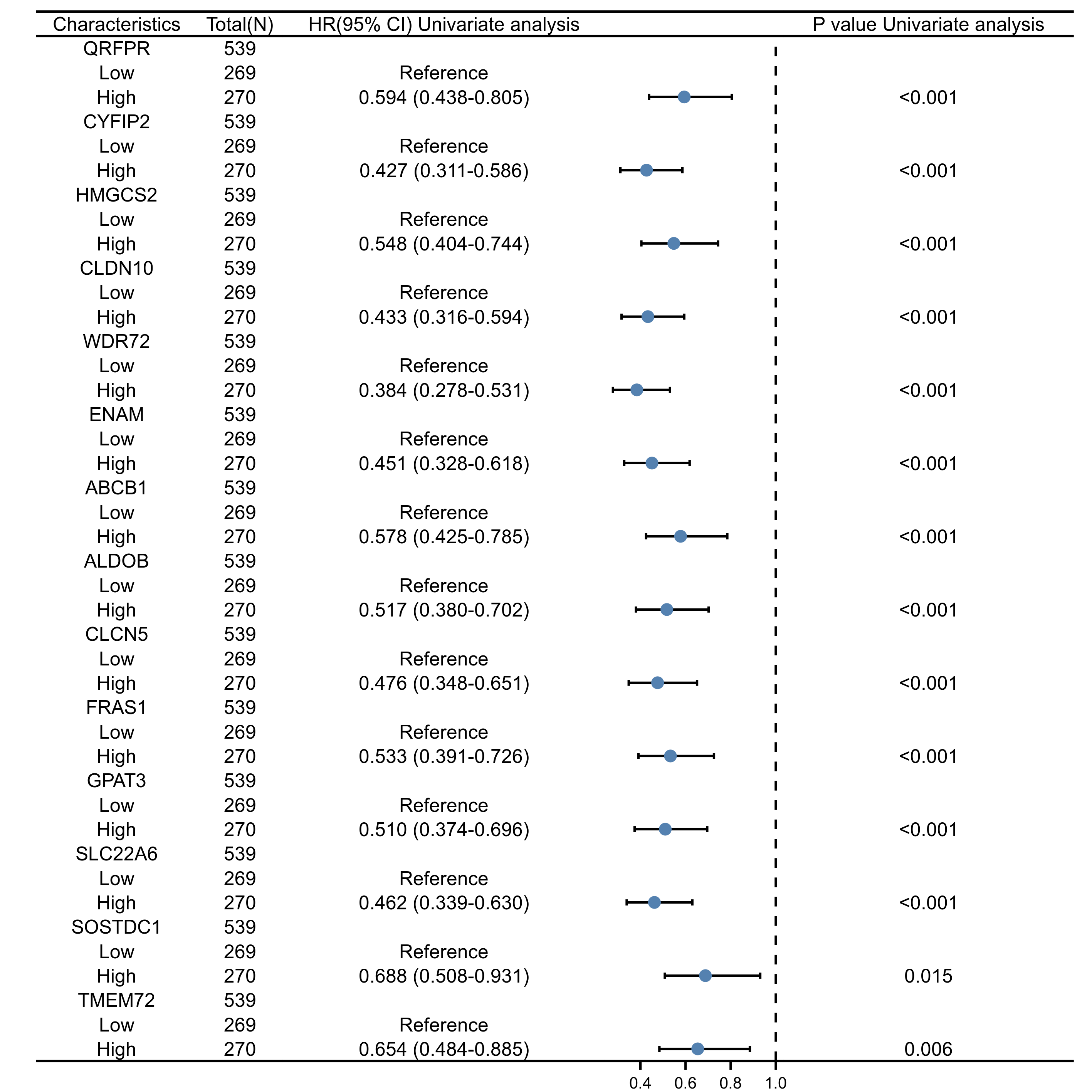

Supplement: Supplementary file 2 [file Image_2.tiff]
