# Supplementary material for: Molecular Subtyping Based on Cuproptosis-Related Genes and Characterization of Tumor Microenvironment Infiltration in Kidney Renal Clear Cell Carcinoma
Source: Front Oncol. 2022 Jul 6;12:919083. doi: 10.3389/fonc.2022.919083 (PMC9299088; doi:10.3389/fonc.2022.919083)
Supplement: Supplementary file 3 [file Image_3.pdf]

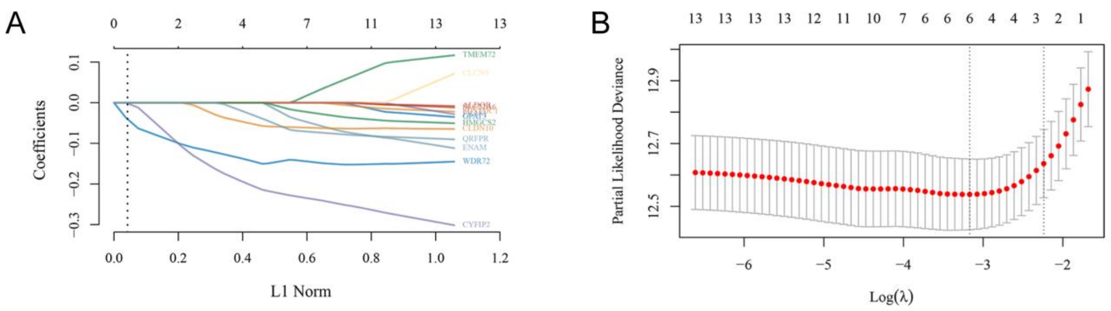

Fig. S3 Construction of a 6-gene signature model in the TCGA cohort. a.LASSO coefficient profiles of the expression of 12 candidate genes. b.Selection of the penalty parameter (λ) in the LASSO model via 10-fold cross-validation. The dotted vertical lines are plotted at the optimal values following the minimum criteria (left) and “one standard error” criteria (right).
