# Supplementary material for: Molecular Subtyping Based on Cuproptosis-Related Genes and Characterization of Tumor Microenvironment Infiltration in Kidney Renal Clear Cell Carcinoma
Source: Front Oncol. 2022 Jul 6;12:919083. doi: 10.3389/fonc.2022.919083 (PMC9299088; doi:10.3389/fonc.2022.919083)
Supplement: Supplementary file 4 [file Image_4.pdf]

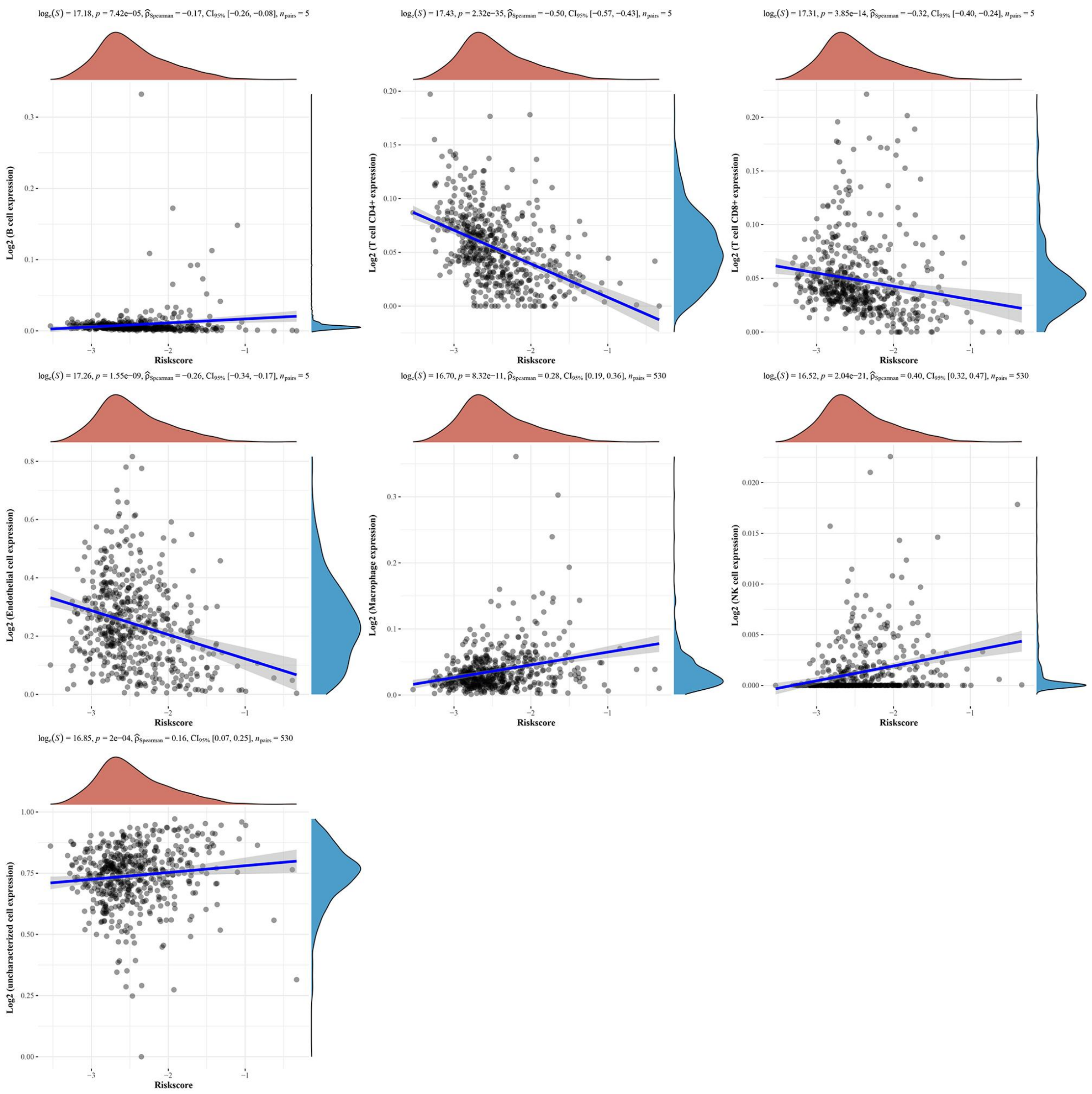

Fig.S4.1 The correlation of CRG_score with immune cells was analyzed using EPIC

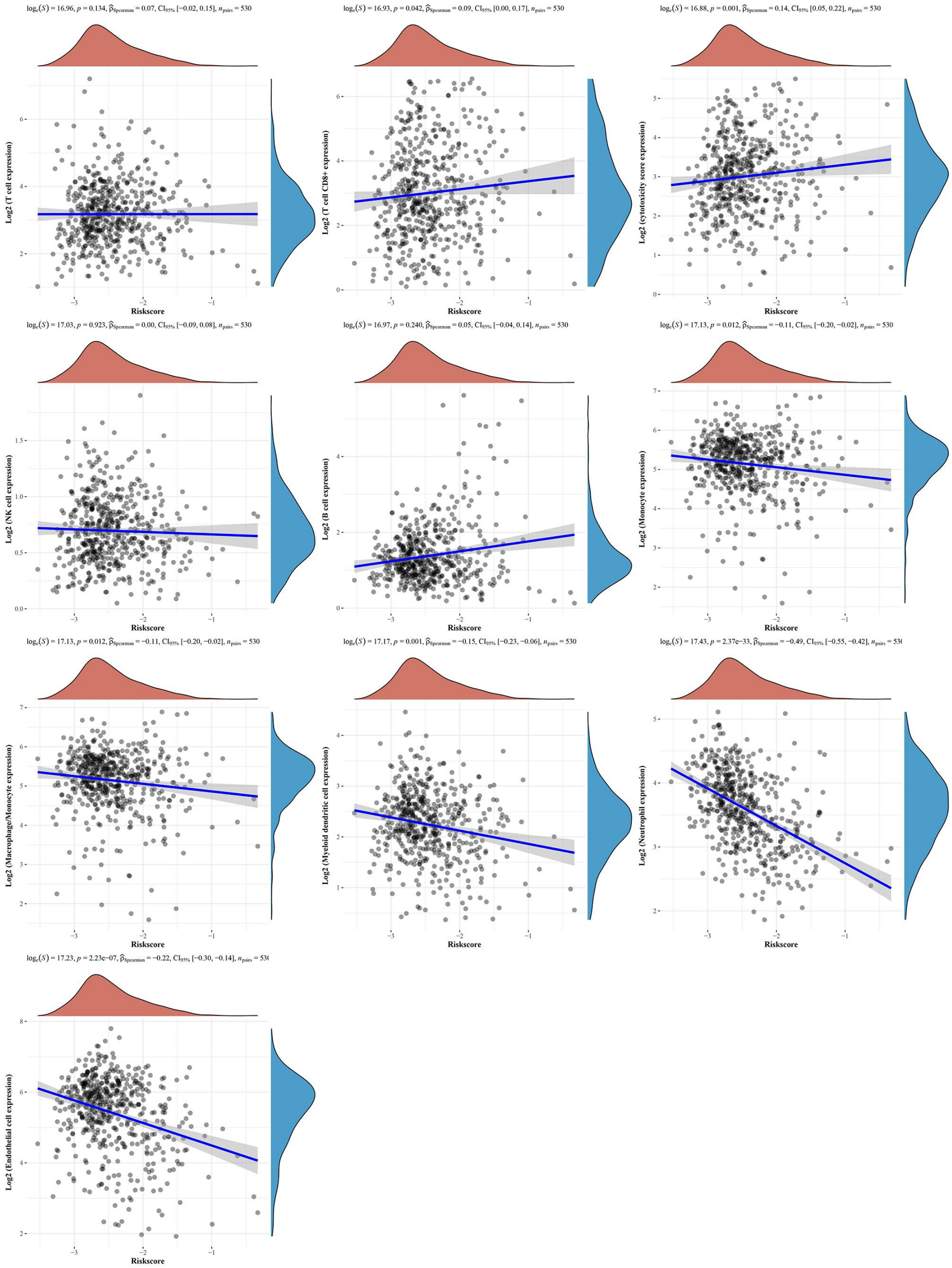

Fig.S4.2 The correlation of CRG_score with immune cells was analyzed using MCP-COUNTER

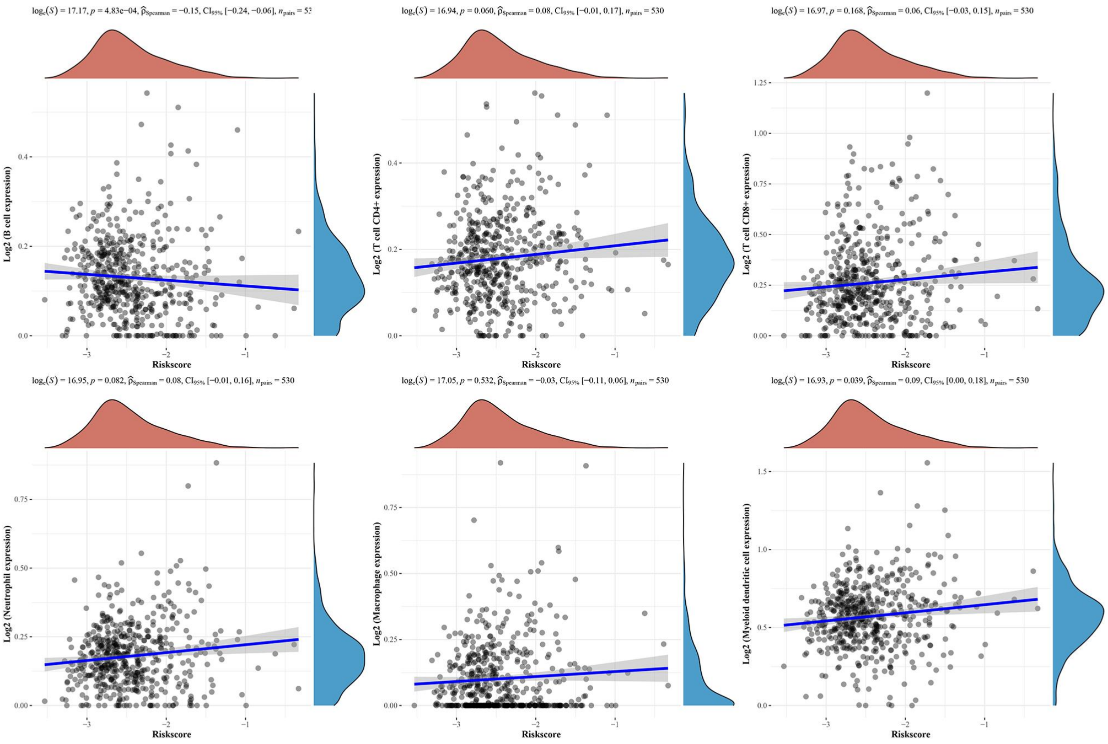

Fig.S4.3 The correlation of CRG_score with immune cells was analyzed using TIMER
